# Anti-*Staphylococcus aureus* Single-Chain Fragment Variables Play a Protective Anti-Inflammatory Role In Vitro and In Vivo

**DOI:** 10.3390/vaccines9111300

**Published:** 2021-11-09

**Authors:** Lei Zhang, Xin Ye, Yan Zhang, Fengqing Wang, Fanqing Zhang, Yan Jia, Dangjin Wu, Kalbinur Tohti, Manling Cheng, Jianguo Zhu

**Affiliations:** 1 Shanghai Key Laboratory of Veterinary Biotechnology, School of Agriculture and Biology, Shanghai JiaoTong University, 800 Dongchuan Road, Shanghai 200240, China; barcelonazl@sjtu.edu.cn (L.Z.); wangfq@sjtu.edu.cn (F.W.); zero0fan@gmail.com (F.Z.); 19981225@mail.sjtu.edu.cn (D.W.); kalbinur@sjtu.edu.cn (K.T.); chengmanling1113@sjtu.edu.cn (M.C.); 2 Laboratory of Regeneromics, School of Pharmacy, Shanghai Jiao Tong University, 800 Dongchuan Road, Shanghai 200240, China; 119170910027@sjtu.edu.cn (X.Y.); jiayan19@sjtu.edu.cn (Y.J.); 3 Key Laboratory of Animal Parasitology of Ministry of Agriculture, Laboratory of Quality and Safety Risk Assessment for Animal Products on Biohazards (Shanghai) of Ministry of Agriculture, Shanghai Veterinary Research Institute, Chinese Academy of Agricultural Sciences, Shanghai 200240, China

**Keywords:** bovine mastitis, *Staphylococcus aureus* (*S. aureus*), single-chain fragment variable (scFv), bovine mammary epithelial (MAC-T) cells, cytotoxicity, anti-inflammatory

## Abstract

*Staphylococcus aureus* is a causative agent of bovine mastitis, capable of causing significant economic losses to the dairy industry worldwide. This study focuses on obtaining single-chain fragment variables (scFvs) against the virulence factors of *S. aureus* and evaluates the protective effect of scFvs on bovine mammary epithelial (MAC-T) cells and mice mammary gland tissues infected by *S. aureus*. After five rounds of bio-panning, four scFvs targeting four virulence factors of *S. aureus* were obtained. The complementarity-determining regions (CDRs) of these scFvs exhibited significant diversities, especially CDR3 of the VH domain. In vitro, each of scFvs was capable of inhibiting *S. aureus* growth and reducing the damage of MAC-T cells infected by *S. aureus*. Preincubation of MAC-T cells with scFvs could significantly attenuate the effect of apoptosis and necrosis compared with the negative control group. In vivo, the qPCR and ELISA results demonstrated that scFvs reduced the transcription and expression of Tumor Necrosis Factor alpha (TNF-α), interleukin-1β (IL-1β), IL-6, IL-8, and IL-18. Histopathology and myeloperoxidase (MPO) results showed that scFvs ameliorated the histopathological damages and reduced the inflammatory cells infiltration. The overall results demonstrated the positive anti-inflammatory effect of scFvs, revealing the potential role of scFvs in the prevention and treatment of *S. aureus* infections.

## 1. Introduction

*Staphylococcus aureus* refers to a natural inhabitant of mammalian skin and mucous epithelia bacterium, acting as the leading causative agent of various diseases in man and domestic animals [1]. The organism accounts for countless diseases, from the skin and soft tissue infections to more invasive diseases (e.g., necrotizing pneumonia and sepsis, especially bovine mastitis) [2]. *S. aureus* is largely involved in intramammary infections (IMI) of lactating cow, which causes significant economic losses to the dairy industry worldwide [3]. After invading the udder via the nipple duct, *S. aureus* can recognize mammary epithelial and inflammatory cells by secreting a series of related toxins and adhesion factors, destroying the integrity of the cell membrane through the joint action of other virulence factors; then, the microbes invade into the cells, causing damage of mammary gland tissues and inflammation [4,5]. The following four proteins (fibronectin binding protein A (FnBPA), coagulase (Coa), β-hemolysin (Hlb), glyceraldehyde-3-phosphate dehydrogenase (GapC)), as important virulence factors, play a vital role in the pathogenicity and inflammation of *S. aureus*. FnBPA can mediate the binding of *S. aureus* to fibrinogen and fibronectin on the surface of host cells, which contributes to the invasion of *S. aureus* to the host cells [6]. Another study showed that the N-terminal of the A domain of FnBPA mediates its binding to fibrinogen and elastin [7]. Coa can make the plasma coagulate by catalyzing the conversion of soluble fibrinogen to insoluble fibrin [8]. The insoluble fibrin wrapped on the surface of the bacteria can protect *S. aureus* from being destructed by bactericidal substances in the serum as well as from the phagocytosis of macrophages. Hlb can destroy the integrity of cell membrane by hydrolyzing lecithin and sphingomyelin, thereby causing cell lysis and hemolysis [9]. Some studies also showed that Hlb can destabilize the cell membrane in lung epithelial cells [10]. GapC is a conserved protein with glyceraldehyde-3-phosphate dehydrogenase (GAPDH) activity. As indicated from several studies, GapC protein participates in the adhesion to MAC-T cells and significantly impacts the pathogenesis of *S. aureus*-induced mastitis [11]. These research of virulence factors contribute to the clarification of disease pathogenesis [12].

The existing antibiotic therapy remains a mainstay for treating bovine mastitis. However, the emergence of antibiotic-resistant bacterial strains and environmental concerns have restricted the use of antibiotics in economic animals. In addition, antimicrobial resistance is an increasingly serious issue in dairy farm, remaining a global public health problem [13]. Some medicinal herbs were demonstrated to exhibit a good therapeutic response to the treatment of bovine mastitis. However, the complexity and side-effects of the components in medicinal herbs while treating bovine mastitis remain unclear [14].

Available vaccines against bovine mastitis infected by *S. aureus* do not provide sufficient protection, despite certain prevention effects. Thus, genetically engineered antibodies are needed which act as an alternative strategy to prevent bovine mastitis [15,16]. According to the data studied previously, injection of antigen-specific egg yolk immunoglobulin (IgY) can ameliorate milk quality and decrease bacterial counts of milk in clinical and experimental cases of bovine mastitis induced by *S. aureus* [17]. Accordingly, the way of passive immunization with antibodies generated against *S. aureus* is very likely to offer an effective means for the prevention and treatment of *S. aureus* infections [18]. The single chain fragment variable (scFv) was deemed one of the most popular types of genetically engineered antibodies, which consists of a variable region of light chains (VL) and heavy chains (VH). The regions of VL and VH associated with antigen binding sites are connected by a short peptide linker. The complete antigen binding sites of the full-length antibody are retained in scFv. ScFv is characterized by its low immunogenicity, strong specificity, small size, and the ability to be genetically engineered. Moreover, scFv can be largely generated in bacterial expression systems [19]. scFv is a powerful tool to prevent and treat microbial diseases; which has already been demonstrated as a treatment to control bacterial and virus infections (e.g., scFv can act on the infections of *Xanthomonas citri* and *Salmonella enteritidis*, and prevent the invasion of chicken infectious bursal disease virus and human influenza virus H5N1) [20,21].

Phage surface display technology is the fusion expression of specific peptides and phage capsid proteins on the phage surface, and the displayed peptides can maintain a relatively independent spatial structure for the recognition and binding of target molecules [22]. As reported, the phage specifically binding to the target molecule was significantly enriched after 3–5 rounds of “adsorption-elution-amplification” [19]. The phage display technology is a useful tool to further enrich scFvs targeting bacterial antigens and secretion (e.g., adhesin and toxin protein). Also, it is a cheaper and more rapid process to generate scFv than hybridoma technology that produces scFv from monoclonal antibodies [23]. At present, the use of phage libraries expressing mouse and human antibody fragments almost became routine in the respective field of biomedical research. Over the past few years, the application of phage display technology in livestock research has increased (e.g., phage display single chain antibody library generated from camels [24], rabbit [25], pig [26], sheep [27], and chicken [28]). However, only a few studies obtained scFvs by phage surface display technology and proved the protective effects of scFvs on bovine mastitis and piglet epizootic diarrhea. Wang et al. reported that scFvs targeting the whole-cell antigen could effectively inhibit the growth of *S. aureus* in vitro and weaken the damage of mammary gland tissues infected by *S. aureus* in vivo [19]. Zhang et al. also reported that scFvs targeting PEDV could neutralize PEDV infection and provide protective efficiency in piglets against PEDV challenge [29].

Proinflammatory cytokine is one of the important indicators of inflammatory response and plays a key role in host defense against invasive pathogenic microorganisms [30]. TNF-α is an “early” multifunctional proinflammatory cytokine that can induce the cascade release of other inflammatory cytokines, such as IL-6 [31]. As a multifunctional cytokine, IL-6 plays a role in regulating inflammation and immune response. As indicated by the data, bovine mastitis has a higher level of IL-6 in milk [32]. IL-1β can regulate *S. aureus*-induced host immune response and recruit inflammatory cells, such as neutrophils, into the lesion area, thus causing a series of inflammatory response [33]. IL-8 and IL-18 are the proinflammatory molecules mediated by NFκB signal pathway in an inflammatory microenvironment. Previous studies showed that IL-8 and IL-18 contributed to neutrophil chemotaxis, which plays an important role in antibacterial immune response [34,35,36]. Inhibiting the production of TNF-α, IL-18, IL-6, IL-1β, and IL-8 is helpful to prevent inflammation. Appropriate levels of proinflammatory cytokines are important for the immune response against pathogens, but excessive production of cytokines may lead to severe tissue damage [37].

In the present study, the binding characteristics of scFvs against virulence factors related to inflammation were evaluated, and the bacterial growth inhibition assay was performed with scFvs. The protective properties of scFvs on MAC-T cells and mice mammary gland tissues infected by *S. aureus* were further confirmed. These results provide basis for the development of scFv-based therapeutic agents, and the mentioned novel bovine scFvs may be suitable candidates to prevent and treat *S. aureus*-induced bovine mastitis.

## 2. Materials and Methods

### 2.1. Bacterial Strains and Cell Culture

Five *S. aureus* strains applied here were termed as USA300 (human-associated *S. aureus* strain kindly provided by professor Jianhe Sun, Shanghai Jiao Tong University, China), ATCC25923 and ATCC29213 (standard *S. aureus* strains preserved in our lab), XD69 and XD79 (cow-associated *S. aureus* strains isolated from Shanghai Xi Di dairy farm, China). *S. aureus* strains were cultured aerobically in triplicate for different hours in Tryptic Soy Broth (TSB) with shaking at 37 °C depending on experiment needs. The supernatants of *S. aureus* were produced through centrifugation, sterilized with 0.22 μm filters, and then stored at −20 °C until use. MAC-T cells (kindly provided by professor Jianhe Sun, Shanghai Jiao Tong University, China) were maintained at 37 °C with 5% CO_2_ in RPMI 1640 supplemented with 10% fetal bovine serum (FBS), no penicillin and streptomycin were added during incubation with *S. aureus* unless stated otherwise.

### 2.2. Preparation of S. aureus Virulence Factors

The genomic DNA of *S. aureus* ATCC25923 strain were extracted with boiling method and served as template to amplify four genes (coa, hlb, gapC and fnbpA). The primers used to amplify the gene sequence of four virulence factors was designed by our lab (Table 1). The underlined nucleic acid sequence is the restriction site that binds to the specific restriction enzyme. The PCR products of the four genes were subcloned into the pET-32a (+) plasmid via double enzyme digestion. Subsequently, the recombinant plasmid was transformed into BL21 (DE3) competent *Escherichia coli* cells (Invitrogen, Carlsbad, CA, USA) [38]. Four hundred milliliters of LB cultures were incubated at 37 °C, until the cultures reached an OD_600_ of 0.6. Next, the cultures were induced with 0.6 mM IPTG (Sigma–Aldrich, St. Louis, MO, USA) at 16 °C, with shaking for 16 h. After centrifugation at 6000× *g* for 20 min, the bacterial pellets were resuspended with phosphate buffered saline (PBS) and then sonicated on ice for 40 min. Afterwards, cell lysates were clarified through centrifugation at 12,000 rpm for 25 min [39]. The soluble proteins with N-terminal 6× His tag were purified with His-Bind Resin (Merk–Novagen, Darmstadt, Germany). The proteins were eluted with 50, 100, 150, 200, 250, 300 mM imidazole contained successively. The protein concentrations were measured by the BCA Protein Assay Kit (Sangon Biotech, Shanghai, China) [19]. The proteins were stored at -80 °C until use.

### 2.3. Optimization and Bio-Panning of the ScFv Phage Display Library

The scFv phage display library was already constructed by Wang et al. in our lab [19]. To obtain a phage library with higher positive rate, we optimized the scFv phage library. Firstly, four virulence factor proteins of *S. aureus* were mixed and coated on a 96-well plate. After five rounds of enrichment and screening of the phage library, a secondary library with higher positive rate was generated. Subsequently, we used the single virulence factor as the coated antigen. The process of the enrichment and the screening can be referenced from previous research conducted in our lab [19,29].

### 2.4. Affinity Selection against Virulence Factors

Phage ELISA was adopted to assess the binding affinity of phages with virulence factor antigens. The bacterial clones were randomly selected for the virulence factor antigens binding analysis after five rounds of panning. Each clone infected by the M13KO7 helper phage was precipitated as described in the previous section. During the screening process of phage ELISA, M13KO7 helper phage was used as the negative control. The description of the procedures performed next can be referenced from previous studies [19,43].

### 2.5. Expression and Purification of ScFvs

To verify the interaction of antigens and scFv in vivo and in vitro experiment, the gene fragment of four scFvs, namely ZL4, ZL15, ZL26, and ZL125 (targeting Hlb, Coa, GapC and FnBPA-A, respectively), were subcloned into pGEX-4T-1 vector carrying N-GST tag. The primers and restriction sites of the four scFvs were identical as below: forward primer named VL-*Eco*RI-F (5′-CCGGAATTCATGGCCCAGGCTGTGCTGACTCAG) and reverse primer named VH-*Xho*I-R (5′-CCGCTCGAGACTAGTGGAGGAGACGGTGAC). The template of the constructed scFv was amplified with the phagemid DNA of positive clones. The subsequent expression and purification of four scFv proteins can be referenced from previous studies [44]. Four soluble scFv proteins with N-GST tag were purified with the GST-Sefinose ™ Kit (Sangon Biotech, Shanghai, China). Afterwards, the proteins were stored at −80 °C until use.

### 2.6. Adhesion and Invasion of S. aureus on MAC-T Cells

RPMI-1640 medium supplied with 10% FBS was used to culture MAC-T cells at 37 °C in 5% CO_2_. Specific to the adhesion assay, 1 × 10^5^ MAC-T cells per well were incubated with *S. aureus* or plus with scFvs (final scFv or mixture concentration was 40 μg/mL in the medium) at a multiplicity of infection (MOI) of 10. After incubation for 3 h, the plates were washed with PBS three times to eliminate bacterial not adhered to MAC-T cells. Next, the cells in every well were lysed with PBS containing 0.1% Triton X-100 for 5 min. The adhered *S. aureus* through the serial dilutions were incubated on TSA plates at 37 °C overnight. Specific to the invasion assay, 1 × 10^5^ MAC-T cells per well were incubated with *S. aureus* or plus with scFvs (final scFv or mixture concentration was 40 μg/mL in the medium) at a MOI of 10. After the cell incubation for 3 h and washing with PBS three times, the medium was substituted with RPMI 1640 medium supplied with 1% FBS and 50 μg/mL of gentamicin (aiming to kill the extracellular *S. aureus*), and then incubated at 37 °C for 1 h. Afterwards, the intracellular invading *S. aureus* were released from the MAC-T cells lysed with PBS containing 0.1% Triton X-100 at 37 °C for 5 min. The adhesion/invasion rate was calculated as (number of CFU of the adhered or invading bacteria/ number of CFU of the initial number of bacteria) × 100% [45].

### 2.7. Bacterial Growth Inhibition Assay

To demonstrate the effect of scFvs aiming at various virulence factors, scFv ZW88 obtained in the previous study was used as the positive control in the bacterial growth inhibition assay. The specific steps of the inhibition assay are as follows. 50 μL *S. aureus* diluted to 10^6^ cfu/mL with TSB were added to a 96-well plate, combined with 50 μL purified scFv (or mixture of four scFvs diluted with TSB), and then added with 100 μL of TSB (final scFv or mixture concentration was 40 μg/mL) with shaking at 37 °C. The mixture of 150 μL of TSB and 50 μL 10^6^ cfu/mL *S. aureus* was set as the negative control. Moreover, the mixture of 150 μL of TSB and 50 μL 10^6^ cfu/mL *S. aureus* containing 40 μg/mL penicillin were set as the positive control. Next, an ELISA plate reader was used to measure the OD_600_ at 6-h intervals [19].

### 2.8. Assessment of Apoptosis by Immunofluorescence Assay (IFA)

For the apoptosis assay, YO-PRO-1 reagent can be used as a sensitive indicator of the apoptosis [46]. Continuously increasing green fluorescence alone indicates apoptotic cells, while the simultaneous red and green fluorescence indicates dead cells. Here, we use YO-PRO^TM^-1/ Propidium Iodide (PI) (Vybrant Apoptosis Assay kit 4; Thermo Fisher, Waltham, MA, USA) to assess the apoptosis of *S. aureus*-induced MAC-T cells. In brief, 1 × 10^5^ MAC-T cells per well was seeded in 96-well culture plates, then washed three times and infected by 1 × 10^6^ cfu/mL of *S. aureus* (MOI = 10) or plus with scFvs (final scFv or mixture concentration was 40 μg/mL) at a range of time points. Next, 100 μL of standard staining working solution was added to the respective well in darkness at 37 °C for 20 min. After being dyed, the stained MAC-T cells were visualized under the epi-fluorescence microscope (EVOS FL-2, Life technologies, New York, NY, USA) [47].

### 2.9. LDH Cytotoxicity Assay

Since the LDH concentration in cell culture media is an indicator of cellular cytotoxicity, the assay can be adopted to monitor cytotoxicity from the identical sample. The level of LDH released in the cell culture supernatant was determined with the LDH Assay Kit (Abcam, Cambridge, UK) in line with the manufacturer’s instructions. To perform the assay, 1 × 10^5^ cells per well were seeded in 96-well culture plates. After being washed with PBS three times, the cells were then infected by 1 × 10^6^ cfu/mL of *S. aureus* (diluted with 1% FBS–RPMI 1640 medium) or plus with scFvs (final scFv or mixture concentration was 40 μg/mL) over time. The negative controls consisted of 200 μL of 1% FBS–RPMI 1640 medium. After the predetermined time points, the 96-well culture plate was centrifuged at 400× *g* for 5–10 min. Subsequently, 120 μL aliquot of the cell culture media was transferred to a novel plate, then, 60 μL detection kit reaction mixture was added to the plate. After a 30-min incubation at the ambient temperature without light, the absorbance was measured with a microplate reader at 490 nm. The cytotoxicity rate was calculated in accordance with the specification [48].

### 2.10. Cell Viability Assay

Cell viability assays were performed by using the CellTiter-Glo^®^ Luminescent Cell Viability Assay (Promega, Madison, WI, USA) according to the manufacturer’s instruction. 1 × 10^5^ MAC-T cells per well was seeded in 96-well culture plates. Subsequently, the supernatant of different *S. aureus* strains or plus with scFvs (final scFv or mixture concentration was 40 μg/mL) were added to the plates at various time points when MAC-T cells grew to about 90%. RPMI-1640 medium was added to the plate as the negative control. After the MAC-T cells were lysed by CellTiter Glo reagent in 10–30 min incubation at ambient temperature, the relative cell viability was determined by luminescence assay based on adenosine triphosphate quantification (CellTiter-Glo). Details of the specific operation steps and calculation methods are in accordance with the specification [49].

### 2.11. Animal Experiment

In the present study, 132 adult female BALB/c mice (6–8 weeks old) that had delivered their first litter of pups 10–15 days in advance were randomly allocated into the control group (C), negative control group (NC), positive control group (PC) and scFv treatment groups (including ZL4, ZL15, ZL26, ZL125, and scFvs mixture group) for intramammary bacterial challenge. Each group was infected by three *S. aureus* trains (USA300, ATCC25923 and XD69). The mastitis model was established as described previously [19]. First, 100 μL of physiological saline were injected subcutaneously into the teats of the R4 and L4 of the mammary gland tissues in C and NC groups. The PC group had 100 μL of 100 mg/mL penicillin injected, while the scFv group injected single scFv or the mixture of four scFvs (20 mg/Kg). Six hours later, 100 μL of physiological saline was injected into the teats of the C group. Meanwhile, the NC, PC and scFv treatment groups had 100 μL of 10^8^ cfu/mL *S. aureus* (USA300, ATCC25923, and XD69) injected. After sodium pentobarbital anesthetized (40 mg/kg), all mice were euthanized with CO_2_ inhalation at 48 h post-inoculation. Subsequently, the blood samples and mammary gland tissues were collected for analysis of the following experimental indicators.

### 2.12. Bacteria Count of Mammary Gland Tissues

Bacterial burden assay was performed to evaluate the inflammatory index. One hundred mg aliquot of mammary gland tissues were homogenized in PBS and then plated onto tryptic soy agar (TSA) plates to enumerate cfu through serial dilutions. Bacterial burden assay was performed with groups of six mice [50].

### 2.13. Histopathological Evaluation of Mammary Gland Tissues

Severe infiltration of neutrophils and macrophages occurs in most fields and areas of mammary gland tissues caused by *S. aureus*. Histopathological evaluation of mammary gland tissues reflects the treatment effect of the scFvs. The mammary gland tissues were excised and fixed with 4% paraformaldehyde, dehydrated, and embedded with paraffin wax. Afterwards, 4 μm thick slices were stained with hematoxylin and eosin (H & E) and observed under an optical microscope (Olympus, Tokyo, Japan) [51,52]. The mammary gland tissues alternation index was determined as described previously [53,54], such as the damage level of mammary acini and mammary epithelial cells, the integrity of lobuli mammae, the inflammatory cells infiltration and the thickening of alveolus walls. Each histological characteristic was graded 0 to 5.

### 2.14. MPO Activity Assay

Myeloperoxidase (MPO) exists in polymorphonuclear neutrophils (PMNs, the main components of inflammation and immune response in bovine mastitis). The inflammatory response severity of the parenchymal infiltration of neutrophils and macrophages in mammary gland tissues was assessed by MPO activity. One hundred mg aliquot of mammary gland tissues were homogenized with reaction buffer (volume ratio 1:9) by homogenizer (GentleMACS Dissociator, Miltenyi Biotec GmbH, Bergisch-Gladbach, Germany). The operating steps were in accordance with the manufacturer’s instructions (Nanjing Jiancheng Bioengineering Institute, Nanjing, China). The value of OD_460_ measured with a 96-well microplate reader was used to evaluate the MPO activity [41,42].

### 2.15. ELISA Assay of Cytokine Expression Levels

In this study, the secretion level of five inflammatory cytokines (TNF-α, IL-18, IL-6, IL-1β, IL-8) was used to evaluate the inflammatory response of mammary gland tissues. The different groups of mammary gland tissues were collected and homogenized with PBS on ice, and then the supernatants were collected by centrifugation. The levels of the five inflammatory cytokines were determined by ELISA kits (Multisciences, Hangzhou, China) according to the manufacturer’s instructions [53,55].

### 2.16. qPCR Assay of Cytokine Transcription Levels

The mammary gland tissues were homogenized in PBS, frozen and thawed three times; the supernatants were obtained after centrifugation at 9000× *g* for 20 min. The total RNA was then extracted from the supernatants of the mammary gland tissues by the TRIzol reagent, and the cDNA was generated by using a reverse transcription kit (Invitrogen, CA, USA) according to the manufacturer’s instructions. The specific primers of five cytokines were listed in Table 1 [35,40,41,42]. Quantitative real-time PCR was performed on an ABI 7300 Real-Time PCR Detection System (Applied Biosystems, Foster, CA, USA) in a 20-μL reaction volume. Each sample was assessed in triplicate. The reaction volume and the reaction condition can be referenced from our lab’s previous studies [29]. The results (fold changes) were normalized by GAPDH using the 2^−ΔΔCt^ comparative method [41].

### 2.17. Statistical Analysis

All tests of the statistical analysis were performed on GraphPad Prism 6 software (San Diego, CA, USA) and SPSS 18.0 software (SPSS, Inc., Chicago, IL, USA). The data were expressed as mean ± standard error. Moreover, the intergroup differences were assessed by multiple comparisons of variance (one-way analysis of variance). The differences were considered statistically significant when *p* < 0.05.

## 3. Results

### 3.1. Expression and Purification of Virulence Factors

**Figure 1:**
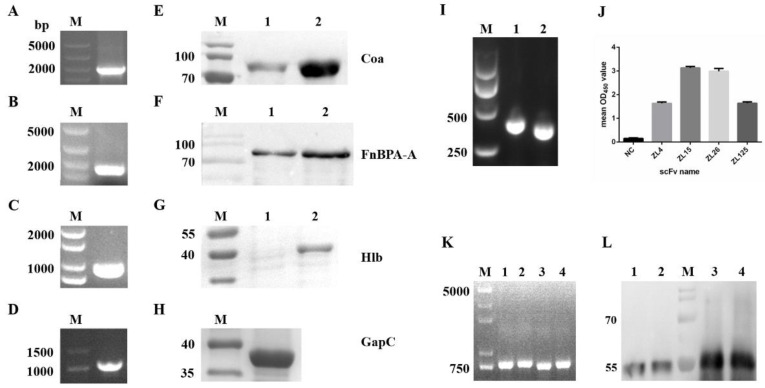
PCR products and protein purification of scFvs and virulence factors of S. aureus. PCR amplification products of coa (**A**), fnbpA-A (**B**), hlb (**C**) and gapC (**D**). Lane M of (**A**–**D**,**I**,**K**), DNA ladder. Western blotting assay of Coa (**E**) and FnBPA-A (**F**), SDS–PAGE analysis of Hlb (**G**), GapC (**H**). Lane M of (**E**–**H**,**L**), protein molecular weight marker; lane 1–2 of (**E**), purified Coa protein eluted by 100 mM imidazole and 150 mM imidazole, respectively; lane 1–2 of (**F**), the purified FnBPA-A protein eluted by 50 mM imidazole and 100 mM imidazole, respectively; lane 1 of (**G**), protein of Hlb without IPTG induction; lane 2 of (**G**), purified Hlb protein eluted by 100 mM imidazole. (**I**), PCR products of VH and VL. Lane 1, PCR products of VH; Lane 2, PCR products of VL. (**J**), Affinity analysis of different scFvs. (**K**), PCR products of scFv amplificated from positive scFv phage clones. lane 1–4, scFv PCR products of ZL4, ZL15, ZL26 and ZL125, respectively. (**L**), Western blotting analysis of four purified scFv products. lane 1–4, purified scFv products of ZL4, ZL26, ZL15 and ZL125, respectively.

Four genes (Figure 1A–D) were subcloned into pET-32a (+) plasmid and then expressed on BL21 (DE3) competent *E. coli* cells. The length of four DNA fragments were about 1000 bp (hlb and gapC), 1600 bp (fnbpA-A), and 2000 bp (coa). Four protein samples were separated by 10% SDS-PAGE and then transferred onto nitrocellulose. The western blotting assay displayed a distinct single band after dyeing, suggesting that the purity of the four antigen proteins was high enough to apply in the following experiment (Figure 1E–H).

### 3.2. The Characteristics and the Screening of ScFv Phage Library

After PCR and overlap PCR, DNA sequences encoding the VL and VH regions were confirmed to be approximately 350 and 380 bp in length, respectively, and the full length of scFv product was around 760–790 bp (Figure 1I,K), including the VL, VH, and (Gly_4_Ser)_3_ linker. The phage display scFv library size was determined as 3.1 × 10^6^ cfu/mL. The positive rate of the library reached over 90% (data not shown). These results revealed the scFv phage library is promising for scFv selection.

After five rounds of bio-panning, the final output titer of the scFv phage display library reached 1.4 × 10^8^ cfu/mL, which was 150-fold higher than the 1st round (Table 2). The positive clone was defined as 2.5-times as much as the OD_450_ of the negative control. Four unique clones targeting the four virulence factors showed a comparatively higher affinity than other positive clones (Figure 1J). These scFvs were designated as ZL4 (accession no. MZ392847), ZL15 (accession no. MZ392848), ZL26(accession no. MZ392849) and ZL125 (accession no. MZ392850) (aiming at Hlb, Coa, GapC and FnBPA-A, respectively).

### 3.3. Identification and Expression of ScFv

Four scFvs carrying N-GST tag were constructed to distinguish the relevant antigens carrying N-6 × His tag. The lengths of the four scFv genes were around 760–790 bp (Figure 1K). All scFvs protein samples were run on 10% SDS-PAGE gels and transferred to nitrocellulose at 150 mA for 1.5 h. As revealed from the SDS-PAGE assay and the Western blots assay, four scFv preteins were approximately 54 kDa (contained 26 kDa of GST tag) in size and displayed the single band, revealing that the purity of the four scFv proteins is satisfactory for the following experiments (Figure 1L).

### 3.4. Characteristics of Purified ScFvs

**Figure 2:**
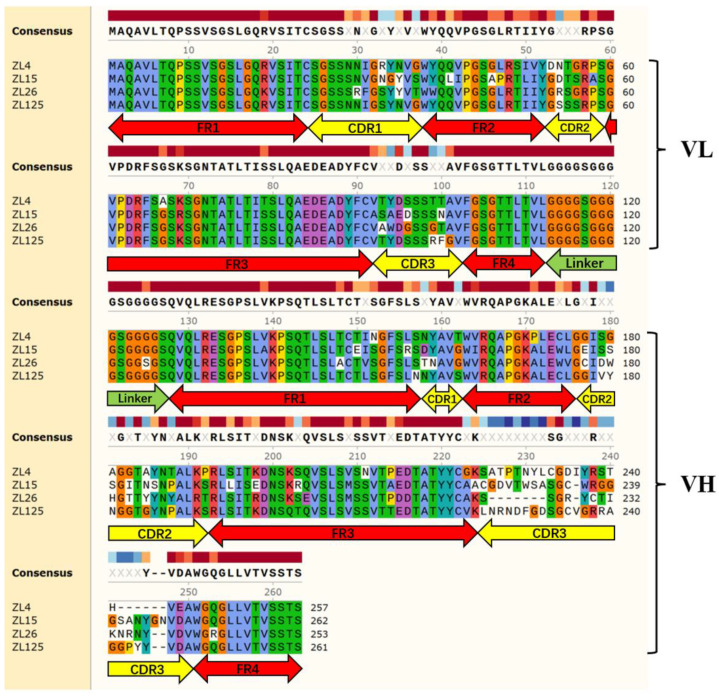
Align multiple protein sequences of ZL4, ZL15, ZL26, and ZL125. Four FRs and three CDRs for both VH and VL of each scFv are illustrated above. Same color regions represent identity of amino acids, while color blank regions represent amino acid differences of four scFvs.

The amino acid sequences of the four scFvs are presented in Figure 2. The VL and VH amino acid sequences of the four scFvs were successively joined with a (Gly_4_-Ser)_3_ peptide linker. Four frame regions (FR) and three complementary determination regions (CDR) constitute VH domain and VL domain, respectively. The FRs of scFv were highly conserved according to amino acid sequencing results. The CDRs of scFv demonstrated a significant diversity, especially CDR3 of VH.

### 3.5. ScFvs Can Affect the Adhesion and Invasion of S. aureus on MAC-T Cells

**Figure 3:**
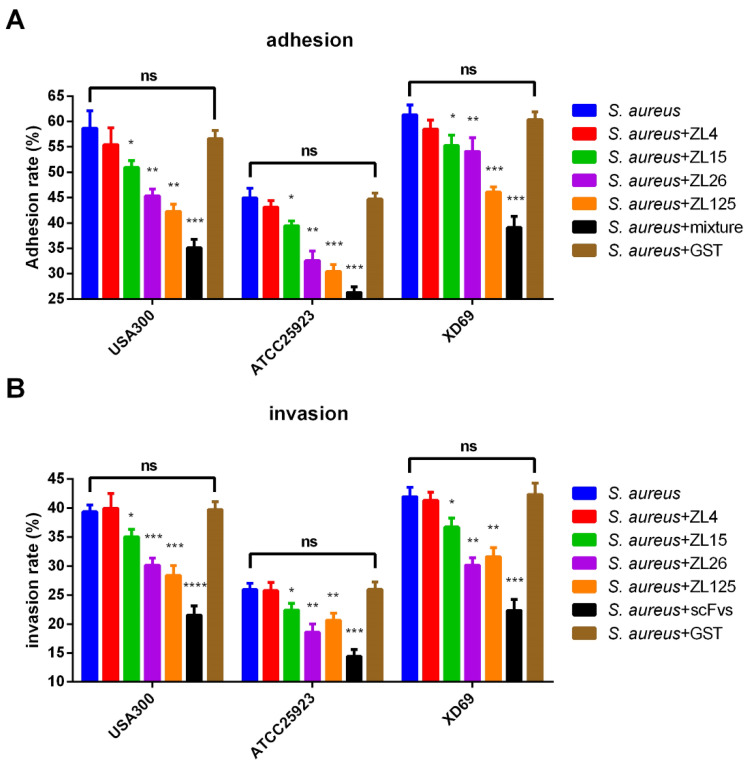
Adhesion (**A**) and invasion (**B**) rate of three *S. aureus* on MAC-T cells or incubated with four scFvs. * *p*, ** *p*, *** *p*, **** *p* vs. the NC group (*S. aureus* group). Data represent mean results ± SD (*n* = 3) (ns = *p* > 0.05, * *p* < 0.05, ** *p* < 0.01, *** *p* < 0.001, **** *p* < 0.0001, *p* vs. the NC group (*S. aureus* group)).

Three *S. aureus* strains USA300, ATCC25923, and XD69 were performed in the adhesion and invasion assay (Figure 3A,B). Specific to the adhesion assay, the adhesion rate of MAC-T cells infected by USA300 and XD69 strains was higher (60%) than that by ATCC25923 strain (44%). After the addition of the mixture of four scFvs, the adhesion rate exhibited a sharp decrease (*p* < 0.001). In the invasion experiment, the invasion rate of USA300, ATCC25923, and XD69 were nearly 39%, 26%, and 42%, respectively. After adding the mixture of four scFvs, the invasion rates were decreased to 21%, 14%, and 22% individually. As suggested from the data, the adhesion rate and invasion rate of MAC-T cells infected by three *S. aureus* strains decreased significantly after adding ZL15, ZL26, ZL125 or the mixture of four scFvs compared with the NC group (*S. aureus* group) (*p* < 0.05), while ZL4 exhibited no effect in adhesion and invasion assay (*p* > 0.05). In addition, no significant difference was found in adhesion and invasion assay between the NC group and the blank control group (*S. aureus* + GST group) (*p* > 0.05).

### 3.6. ScFvs Can Effectively Inhibit the Bacteria Proliferation In Vitro and In Vivo

**Figure 4:**
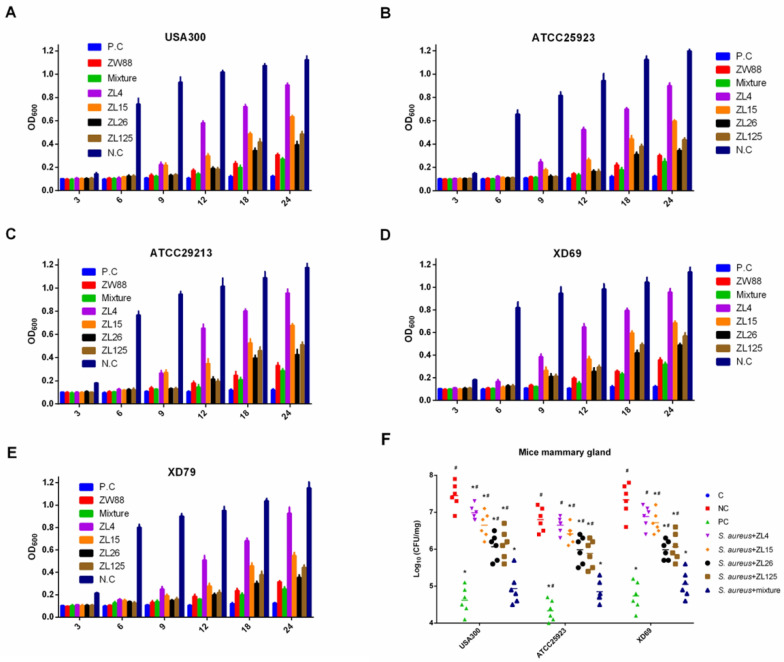
Function of scFvs to growth inhibition of *S. aureus* strains USA300 (**A**), ATCC25923 (**B**), ATCC29213 (**C**), XD69 (**D**), and XD79 (**E**). (**F**) Bacteria proliferation was inhibited by four scFvs in vivo. *S. aureus* strains were cultured in TSB containing physiological saline, scFvs or penicillin (final concentration of both scFv/mixture and penicillin were 40 μg/mL). Data represent mean results ± SD (*n* = 3 or 6). # *p* < 0.05 vs. scFvs mixture group. * *p* < 0.05 vs. negative control group.

The growth of *S. aureus* USA300, ATCC25923, ATCC29213, XD69, and XD79 in TSB were inhibited to some extent by the single scFv (ZL4, ZL15, ZL26, ZL125 and ZW88) or the mixture of four scFvs compared with NC in 24 h (Figure 4A–E). Furthermore, the inhibiting effect of scFvs mixture group was better than that of scFv ZW88 group in five strains. Nevertheless, the supernatant of five *S. aureus* strains incubated with the mixture of four scFvs and scFv ZW88 became turbid in TSB in 18 h, the OD_600_ (about 0.25) of which were less than the NC group (about 1.12). To determine the specific contribution of scFvs to the inflammatory response, we monitored colonization and bacterial replication in mammary gland tissues systemically infected with *S. aureus*. The results showed that the bacterial burden of USA300 and XD69 were higher than that of ATCC25923 in mammary gland tissues. In addition, the effect of the mixture of four scFvs exhibited a 100-fold decrease in bacterial burden compared with the NC group (*p* < 0.05). Furthermore, the scFvs mixture group significantly inhibited the bacteria proliferation compared with the single scFv group at 48 h (*p* < 0.05), which exhibited a 10–100-fold decrease in bacterial burden (Figure 4F). The results showed that scFvs could effectively inhibit the proliferation of *S. aureus* in vitro and in vivo.

### 3.7. ScFvs Can Attenuate the Apoptosis and Necrosis of S. aureus on MAC-T Cells

**Figure 5:**
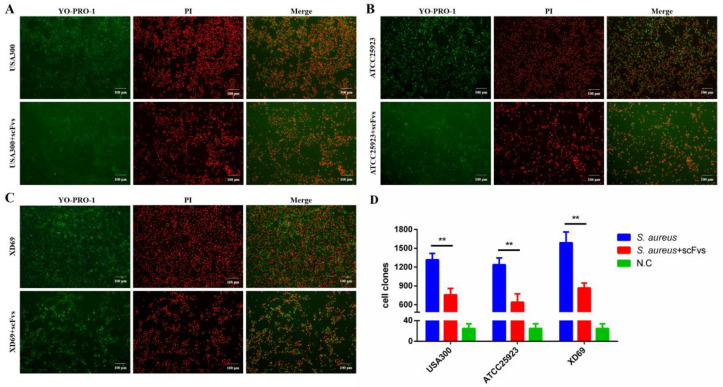
Apoptosis and necrosis of MAC-T cells infected by *S. aureus* or plus with mixture of scFvs. MAC-T cells treated with USA300 (**A**), XD69 (**B**) and ATCC25923 (**C**) strains or plus with mixture of scFvs. Continuously increasing green fluorescence alone indicates apoptotic cells, while simultaneous red and green fluorescence indicates dead. (**D**) Cell clones of apoptotic and necrotic MAC-T cells infected by three *S. aureus* strains. Data represent mean results ± SD (*n* = 3) (** *p* < 0.01).

The apoptosis assay was performed for MAC-T cells infected by USA300, XD69, and ATCC25923, respectively (Figure 5A–C). As revealed from the results, *S. aureus* could cause apoptosis and necrosis of MAC-T cells. After adding the mixture of four scFvs, the number of apoptotic and necrotic MAC-T cells infected by three *S. aureus* strains decreased significantly from 1300 to 750 (USA300 group) (*p* < 0.01), from 1240 to 640 (ATCC25923) (*p* < 0.01), and from 1590 to 860 (XD69 group) (*p* < 0.01) (Figure 5D).

### 3.8. ScFvs Can Weaken the Cytotoxicity of S. aureus on MAC-T Cells

**Figure 6:**
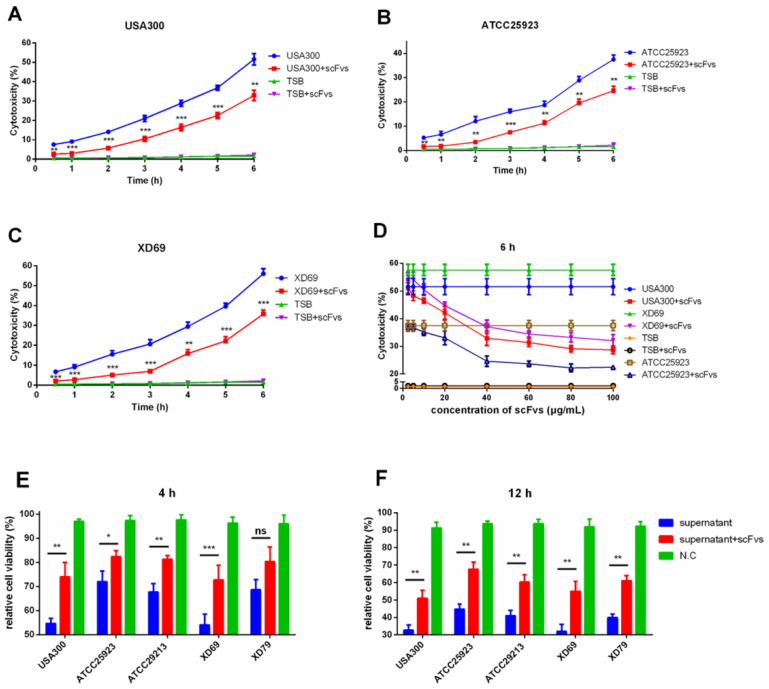
LDH cytotoxicity assay and relative cell viability assay. LDH release of MAC-T cells infected by strain USA300 (**A**), ATCC25923 (**B**), XD69, (**C**) over time. (**D**) LDH release of MAC-T cells after treatment with three *S. aureus* strains and the mixture of various concentrations of scFvs. Relative cell viability of MAC-T cells infected by five *S. aureus* strains in 4 h (**E**) and 12 h (**F**). Data represent mean results ± SD (*n* = 3) (ns = *p* > 0.05, * *p* < 0.05, ** *p* < 0.01, *** *p* < 0.001).

The LDH cytotoxicity assay detection kit was adopted to detect the level of LDH released in the cell culture supernatant. Three *S. aureus* strains (i.e., USA300, ATCC25923, and XD69) were selected to perform the assay. The results revealed that the level of LDH concentrations in the supernatant were continually elevated over time. After the infections for 6 h, the LDH release of MAC-T cells infected by strain USA300 (from 50% to 34%) (*p* < 0.01), ATCC25923 (from 40% to 25%) (*p* < 0.01), and XD69 (from 55% to 37%) (*p* < 0.001) decreased significantly after incubation with the mixture of four scFvs (Figure 6A–C). It is worth noting that the mixed scFvs had a dose-dependent protective effect on MAC-T cells (Figure 6D). Meanwhile, the relative cell viability assay was conducted by detecting the level of ATP release of MAC-T cells infected by the supernatant of USA300, ATCC25923, ATCC29213, XD69, and XD79, showing that the percentage of the relative cell viability of MAC-T cells infected by the supernatant of XD69 and USA300 strains was lower than that of the cells infected by the supernatant of other three strains. The differences between the supernatant of five *S. aureus* and plus with the mixture of scFvs for infections to MAC-T cells showed the statistical significance (*p* < 0.05) (Figure 6E,F). The downtrend of the relative cell viability of MAC-T cells incubated with the mixture of scFvs and the culture supernatants were distinct to varying degrees by the change over time.

### 3.9. Effect of ScFvs on S. aureus-Induced Histopathologic Changes and MPO Activity of Mammary Gland Tissues

**Figure 7:**
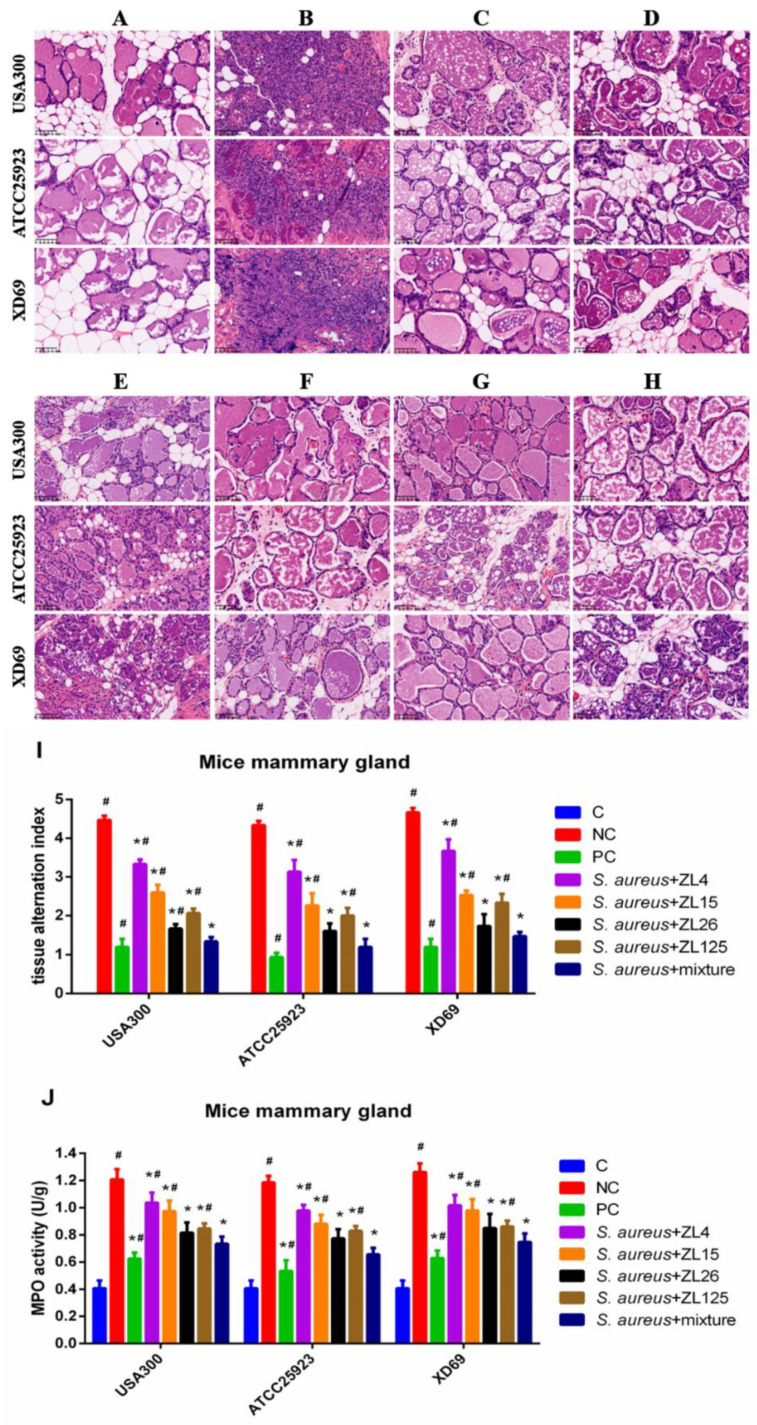
Functions of scFvs on histopathological changes in *S. aureus*-induced mammary gland tissues by H & E staining. (**A**) Control group; (**B**) negative control group; (**C**) positive control group; (**D**) scFvs mixture group; (**E**–**H**) single scFv group (ZL4, ZL15, ZL26 and ZL125, respectively); (**I**) Tissue alteration index in mammary gland tissues; (**J**) MPO activity assay. Red arrow was tissue lesion area (red arrow indicates inflammatory cells infiltration in mammary gland tissues). Data represent mean results ± SD (*n* = 6). # *p* < 0.05 vs. scFvs mixture group. * *p* < 0.05 vs. negative control group.

The pathological section of mammary gland tissues was detected by H & E staining and microscopic observation. The results showed no histopathological changes in the C group (Figure 7A). The mammary gland tissue structures of the NC group treated with three *S. aureus* strains were destroyed, showing severe histopathologic changes such as damaged mammary acini and mammary epithelial cells, incomplete lobuli mammae, and inflammatory cells infiltration (Figure 7B). In contrast, these histological changes of the scFv treatment groups were significantly ameliorated (*p* < 0.05), particularly the scFvs mixture group, the effect of which was close to the PC group exhibiting very slight inflammatory injury and mild inflammatory cells interstitial infiltration (Figure 7C–H). The mammary gland tissues alternation index was used to evaluate the histopathological changes in the mammary gland tissues, such as the integrity of mammary tissues and the numbers of infiltrated inflammatory cells (Figure 7I). Besides, the MPO activity of five scFv treatment groups, including single scFv group and scFvs mixture group, was significantly reduced compared with that of the NC group (*p* < 0.05) (Figure 7J).

### 3.10. Effect of ScFvs on Inflammatory Cytokine Production in Mammary Gland Tissues

**Figure 8:**
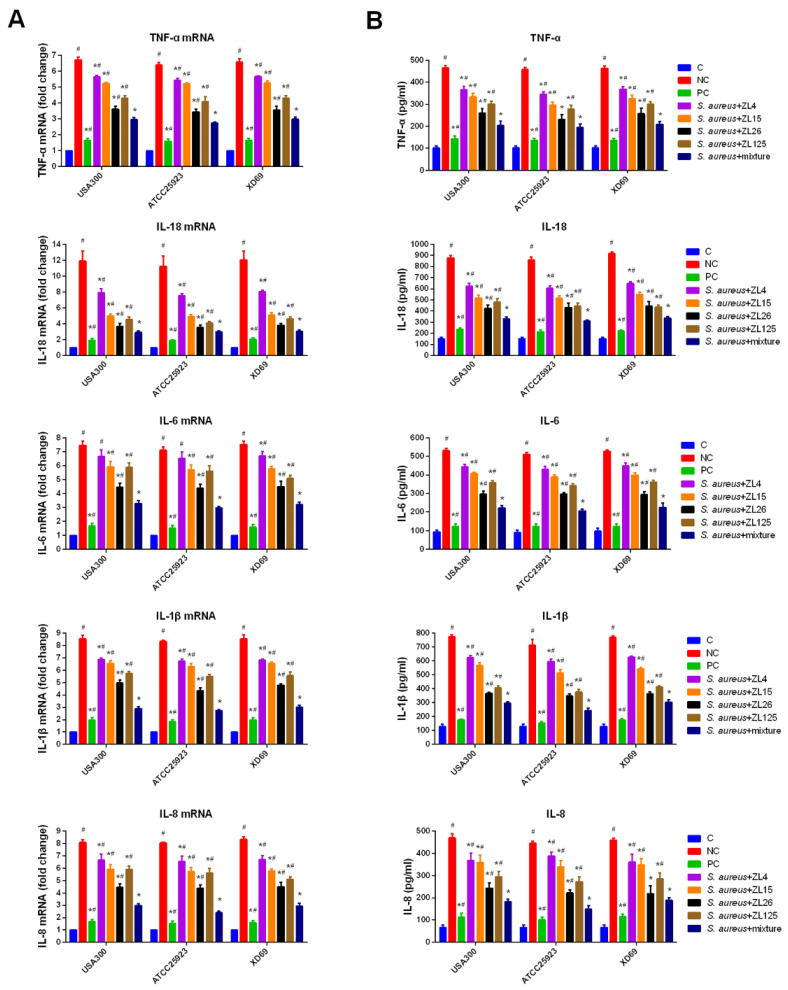
Functions of scFvs on production of proinflammatory cytokines in mammary gland tissues. (**A**) Transcription levels of TNF-α, IL-18, IL-6, IL-1β, and IL-8 detected by qPCR method in mammary gland tissues; (**B**) expression level of TNF-α, IL-18, IL-6, IL-1β, and IL-8 detected by ELISA method in mammary gland tissues. *GAPDH* was used as control gene. Data represent mean results ± SD (*n* = 6). # *p* < 0.05 vs. scFvs mixture group. * *p* < 0.05 vs. negative control group.

To investigate the effects of scFvs on the *S. aureus*-induced inflammatory cytokines production, the levels of TNF-α, IL-18, IL-6, IL-1β, and IL-8 were measured by qPCR and ELISA assays. The results indicated that every single scFv could dramatically reduce the expression levels of five inflammatory cytokines (*p* < 0.05). Compared with that of the NC group, the expression levels of TNF-α (from 480 pg/mL to 190 pg/mL), IL-18 (from 880 pg/mL to 350 pg/mL), IL-6 (from 520 pg/mL to 210 pg/mL), IL-1β (from 770 pg/mL to 300 pg/mL), and IL-8 (from 460 pg/mL to 190 pg/mL) in the scFvs mixture treatment group approximately exhibited a 2.5-fold decrease (*p* < 0.05) (Figure 8B). Besides, the transcription levels of the five inflammatory cytokines in mammary gland tissues infected by three *S. aureus* strains were significantly suppressed by the scFvs mixture group compared with the NC group (*p* < 0.05), exhibiting a 2.5-fold decrease (the fold changes decrease approximately from 6.74 to 2.92 in TNF-α group, from 12.02 to 2.48 in IL-18 group, from 7.22 to 3.16 in IL-6 group, from 8.52 to 2.85 in IL-1β group, and from 8.03 to 2.87 in IL-8 group). Moreover, Almost all of the scFvs mixture groups (except for the expression of TNF-α in ATCC25923 group and IL-8 in XD69 group) showed remarkable inhibitory effects compared with the single scFv group (*p* < 0.05) (Figure 8A,B).

## 4. Discussion

Bovine mastitis is generally known as one of the most prevalent diseases affecting the dairy industry and causing considerable economic losses. *S. aureus* is one of the extremely important causative organisms that can cause bovine mastitis [56]. At present, the enhancement of drug resistance and antibiotic residues have increasingly become important factors endangering public health, despite certain therapeutic effects in the treatment of bovine mastitis. The existing studies demonstrated that the killing effect of *S. aureus* on cells and mice varies significantly with the source of the strains [57]. Previous studies revealed that specific *S. aureus* genotypes (i.e., USA300) showed an increased cow-to-human cross-infection [58]. Therefore, *S. aureus* (five strains) or their culture supernatants were studied to verify the pathogenicity of *S. aureus* from different sources. In brief, four scFvs exhibiting a strong interaction with four virulence factors were obtained by using the phage display technique. The overall results showed that USA300 and XD69 had stronger pathogenicity compared with ATCC25923, ATCC25923, and XD79, and the protective effects of the four scFvs on MAC-T cells and mice mammary gland tissues infected by *S. aureus* was confirmed.

The positive rate of the phage library was improved by optimizing the screening condition, such as reducing the concentration of coated antigen from 10 μg/mL in the first round to 5 μg/mL in subsequent rounds, and increasing the washing times from 10 to 20 times in the subsequent rounds with PBST and PBS. The results revealed that the output/input of the scFv phage display library in the 5th round (1.33 × 10^−5^) is 150 folds higher than that in the 1st round (8.93 × 10^−8^). Previously, Wang et al. reported that the final output titer of the scFv phage display library reached 2.32 × 10^7^ cfu/mL, which is 6 folds lower than in this study (1.4 × 10^8^ cfu/mL) [19,29].

As indicated from the comparison of amino acid sequence, Framework regions (FRs) are highly conserved, while the CDRs of antibody binding sites and the specific determination are highly variable, suggesting that scFvs obtained in this study may well have various epitope specificities. For the composition and the numbers of amino acids, the largest variation was in the CDR3 of VH, suggesting that in the process of binding to antigen, the CDR3 may be the most important antigen recognition site. Future studies should focus on the spatial structure and binding mode of scFv to explore the antigen-antibody interaction mechanism. Also, it will be interesting to study whether the region interacting with the corresponding virulence factors of *S. aureus* belongs to the CDR regions of scFv, especially the CDR3 region of VH.

Compared with that of the NC group, the adhesion rate and invasion rate of USA300, ATCC25923 and XD69 strains decreased in different degrees after incubation with ZL26, ZL125, and the mixture of four scFvs, respectively. The adhesion rate and invasion rate of ZL26 and ZL125 treatment groups decreased significantly compared with that of the NC group (*p* < 0.01), illustrating that the interaction between scFvs (ZL125 and ZL26) and the adhesins (i.e., FnBPA and GapC) secreted by *S. aureus* may be responsible for the weakening of the adhesion of *S. aureus* to MAC-T cells, thus effecting the invasion. Previous studies demonstrated that FnBPA and GapC contributed to the adhesion and invasion of *S. aureus* to the host cells, complying with the results in this study [6,11]. The adhesion rate of USA300 (from 56% to 51% in ZL15 group (*p* < 0.05), and from 56% to 42% in ZL125 group (*p* < 0.01)), ATCC25923 (from 44% to 39% in ZL15 group (*p* < 0.05), and from 44% to 30% in ZL125 group (*p* < 0.001)) and XD69 (from 58% to 54% in ZL15 group (*p* < 0.05) and from 58% to 45% in ZL125 group (*p* < 0.001)) decreased in varying degrees after the interaction with ZL15 (against Coa) and ZL125 (against FnBPA). As reported, Coa could combine with FnBPA to form fibrin agglutination on the staphylococci surface and promote bacterial adhesion [8]. Moreover, scFvs mixture group exhibited a stronger inhibiting effect in adhesion and invasion compared with that of single scFv treatment group. The overall results suggested that scFvs may interact with Coa, FnBPA and GapC, thus blocking the formation of fibrin agglutination and decreasing the adhesive attraction.

As suggested from the apoptosis assay, *S. aureus* could cause apoptosis and necrosis of MAC-T cells, complying with the results of the existing studies [59]. The number of apoptotic cells and necrotic cells in scFv treatment groups decreased approximately by half (from 1300 to 750 in USA300 group (*p* < 0.01), from 1240 to 640 in ATCC25923 group (*p* < 0.01) and from 1590 to 860 in XD69 group (*p* < 0.01), respectively) compared with that of the NC group. Previous studies demonstrated that Panton–Valentine leucocidin and enterotoxin H of *S. aureus* could induce apoptosis of bovine mammary epithelial cells [60,61]. Additionally, Coa, FnBPA, and GapC can affect the adhesion and invasion of *S. aureus* to the host cells [6,8,11], and Hlb can destroy the integrity of cell membrane [9,10]. Therefore, it is speculated that the four virulence factors possibly worked together to destroy the cell membrane, thus leading to apoptosis and necrosis of MAC-T cells. More mechanisms of apoptosis resulted by *S. aureus* need to be investigated in the following research.

LDH acts as a vital index of cell membrane integrity. The concentration of LDH in the culture supernatant of MAC-T cells infected by USA300 (*p* < 0.01), ATCC25923 (*p* < 0.01) and XD69 (*p* < 0.001) in scFv treatment groups decreased significantly compared with the NC group (*p* < 0.01). Wang et al. reported that *S.* *aureus* could lead to nearly 80% of LDH release on A539 cells, the results of which were similar with the present studies [62]. Besides, the mixed scFvs had a dose-dependent protective effect on MAC-T cells. Furthermore, the relative cell viability assay showed that USA300 and XD69 exhibited stronger pathogenicity to MAC-T cells compared with that of other *S. aureus* strains in vitro. The relative cell viability assay also presented similar results with cytotoxicity assay [63]. These studies indicated that the four virulence factors of *S. aureus* can destroy the cell membrane [6,8,9,11]. Moreover, relevant scFvs can inhibit the cytotoxic effects of *S. aureus* on MAC-T cells, thus possibly providing a potential protective effect on dairy cow mastitis caused by *S. aureus*.

A mouse model of *S. aureus*-infected mastitis was established to evaluate the effects of scFvs on the inflammatory response in mammary gland tissues. The results of histopathological changes in the NC group revealed that the mammary acini and mammary epithelial cells were damaged, the lobuli mammae was incomplete, and the inflammatory cells were infiltrated in mammary gland tissues. These results were accordant to the previous studies [53,54]. Comparatively, these histological changes were significantly ameliorated and the MPO activity was significantly reduced in the scFv treatment groups (*p* < 0.05). Moreover, the effect of scFvs mixture group on the decrease of MPO activity was better than that of the single scFv group, indicating that the scFvs probably produce potential anti-inflammatory effects, especially the scFvs mixture.

We detected the production of five proinflammatory cytokines to verify the key role in host defense against invasive pathogenic micro-organisms. The results showed that compared with the C group, the transcription level and expression level of TNF- α, IL-18, IL-6, IL-1β and IL-8 increased significantly in mammary gland tissues infected by *S. aureus*, especially IL-1β and IL-8 (the expression levels reached over 700 pg/mL) (*p* < 0.05). Additionally, MPO activity (from about 1.21 to 0.78) assay also manifested that the neutrophils could be recruited to the inflammation area in the NC group. Similarly, previous studies demonstrated that TNF-α could induce the cascade release of other inflammatory cytokines, such as IL-6 [31]. IL-6 played a role in regulating inflammation and immune response [32]. IL-1β can regulate *S. aureus*-induced host immune response and recruit inflammatory cells, such as neutrophils, into the lesion area, thus causing a series of inflammatory response [33]. In the present study, TNF-α, IL-6, and IL-1β exhibited a sharp decrease in scFvs mixture group compared with the NC group (*p* < 0.05), indicating that scFvs have anti-inflammatory effects on mammary gland tissues. IL-8 and IL-18 contributed to neutrophil chemotaxis, which plays an important role in anti-bacterial immune response [34,35,36]. These studies indicated that suitable levels of five proinflammatory cytokines are vital for host defenses, whereas overmuch production can cause systemic inflammation and damage rather than protective effects. Here in this study, the production of five proinflammatory cytokines in three groups (USA300, ATCC25923 and XD69) exhibited no obvious difference. The results revealed that the transcription level and expression level of five proinflammatory cytokines in scFv mixture group reduced significantly compared with the NC group (*p* < 0.05). In addition, the anti-inflammatory effect in scFv mixture treatment group (except for the expression of TNF-α in ATCC25923 group and IL-8 in XD69 group) was more significant than that of single scFv group (*p* < 0.05). Generally, the overall results indicated that scFvs attenuated the damage of mammary gland tissues in vivo by regulating the transcription level and expression level of five proinflammatory cytokines, which is consistent with the study of Wang et al. [19].

## 5. Conclusions

In summary, four scFvs against different virulence factors were screened by using phage display techniques in this study. These scFvs could significantly inhibit the adhesion and invasion of *S. aureus* to MAC-T cells and exhibited bacteriostatic effects in vitro and in vivo. The scFvs could also reduce the apoptosis and cytotoxicity of *S. aureus* on MAC-T cells. Besides, as proven from a variety of experiments, scFvs have certain protection effects on mammary gland tissues of mice and MAC-T cells infected by *S. aureus*. In the future, in-depth investigations on the anti-inflammatory function of scFvs and the possible molecular mechanisms need to be conducted. The data presented in this study could be useful for further studies on the pathogenic mechanism of virulence factors and ideal drug targets screening, which will assist in the prevention and treatment of dairy cow mastitis attributed to *S. aureus*.

## Figures and Tables

**Table 1:** List of primers used in this study.

Primer Name	Sequence (5′-3′)	Size (bp)	Reference
Coa-F	CGCGGATCCAGCTTATTTACATGGGAT	1932	This study
Coa-R	CCGCTCGAGTTATTTTGTTACTCTAGGC		
Hlb-F	CGCGGATCCGCCGAATCTAAGAAAGATGA	891	This study
Hlb-R	CCGCTCGAGTTTACTATAGGCTTTGATTGGA		
FnBPA-A-F	CCGGGATCCGCATCAGAACAAAAGACAAC	1629	This study
FnBPA-A-R	CCGAAGCTTTATCAATAGCTGATGAATCCG		
GapC-F	CGCGGATCCATGGCAGTAAAAGTAG	1008	This study
GapC-R	CCGCTCGAGTTTAGAAAGTTCAGCTAAG		
IL-18-F	TGGTTCCATGCTTTCTGGACTCCT	132	[40]
IL-18-R	TTCCTGGGCCAAGAGGAAGTGATT		
TNF-α-F	GCCTCCCTCTCATCAGTCTA	223	[41]
TNF-α-R	GGCAGCCTTGTCCCTG		
IL-6-F	AGTTGTGCATGGCAATTCTGA	213	[41]
IL-6-R	AGGACTCTGGCTTGTCTTTCT		
IL-1β-F	ACCTGTGTCTTCCCGTGG	171	[41]
IL-1β-R	TCATCTCGAGCCTGTAGTG		
IL-8-F	CGGCAATGAAGCTTCTGTAT	224	[35]
IL-8-R	CCTTGAAACTCTTTGCCTCA		
GAPDH-F	CAATGTGTCCGTCGTGGATCT	124	[42]
GAPDH-R	GTCCTCAGTGTAGCCCAAGATG		

**Table 2:** Phage library from five rounds of bio-panning.

Round of Screening	Input (cfu/mL)	Output (cfu/mL)	Output/Input	Enrichment Fold	Total Enrichment Fold
1	1.12 × 10^13^	1.00 × 10^6^	8.93 × 10^−8^	1	
2	1.09 × 10^13^	1.58 × 10^6^	1.45 × 10^−7^	1.62	
3	1.15 × 10^13^	3.12 × 10^6^	2.71 × 10^−7^	1.87	
4	1.21 × 10^13^	4.39 × 10^7^	3.63 × 10^−6^	13.39	
5	1.05 × 10^13^	1.40 × 10^8^	1.33 × 10^−5^	3.66	148.93

## Data Availability

The datasets generated and analyzed during the current study are available from the corresponding author on reasonable request.
