# Correction: Zhang et al. Anti-*Staphylococcus aureus* Single-Chain Fragment Variables Play a Protective Anti-Inflammatory Role In Vitro and In Vivo. *Vaccines* 2021, *9*, 1300

**DOI:** 10.3390/vaccines10081351

**Published:** 2022-08-19

**Authors:** Lei Zhang, Xin Ye, Yan Zhang, Fengqing Wang, Fanqing Zhang, Yan Jia, Dangjin Wu, Kalbinur Tohti, Manling Cheng, Jianguo Zhu

**Affiliations:** 1 Shanghai Key Laboratory of Veterinary Biotechnology, School of Agriculture and Biology, Shanghai JiaoTong University, 800 Dongchuan Road, Shanghai 200240, China; 2 Laboratory of Regeneromics, School of Pharmacy, Shanghai Jiao Tong University, 800 Dongchuan Road, Shanghai 200240, China; 3 Key Laboratory of Animal Parasitology of Ministry of Agriculture, Laboratory of Quality and Safety Risk Assessment for Animal Products on Biohazards (Shanghai) of Ministry of Agriculture, Shanghai Veterinary Research Institute, Chinese Academy of Agricultural Sciences, Shanghai 200240, China

In the original publication [1], there was a mistake in **Figure 7. Functions of scFvs on histopathological changes in *S. aureus*-induced mammary gland tissues by H & E staining** as published. We used the wrong picture of the XD69 group in Figure 7D, and this needed to be replaced with the new, correct XD69 picture. It is important to note that the results of the tissue change index in Figure 7I are all correct, it is just that the figure was misplaced. These corrections do not affect the conclusion of this figure nor of the study. The corrected Figure 7 appears below. The authors apologize for any inconvenience caused and state that the scientific conclusions are unaffected. This correction was approved by the Academic Editor.

## Figures and Tables

**Figure 7:**
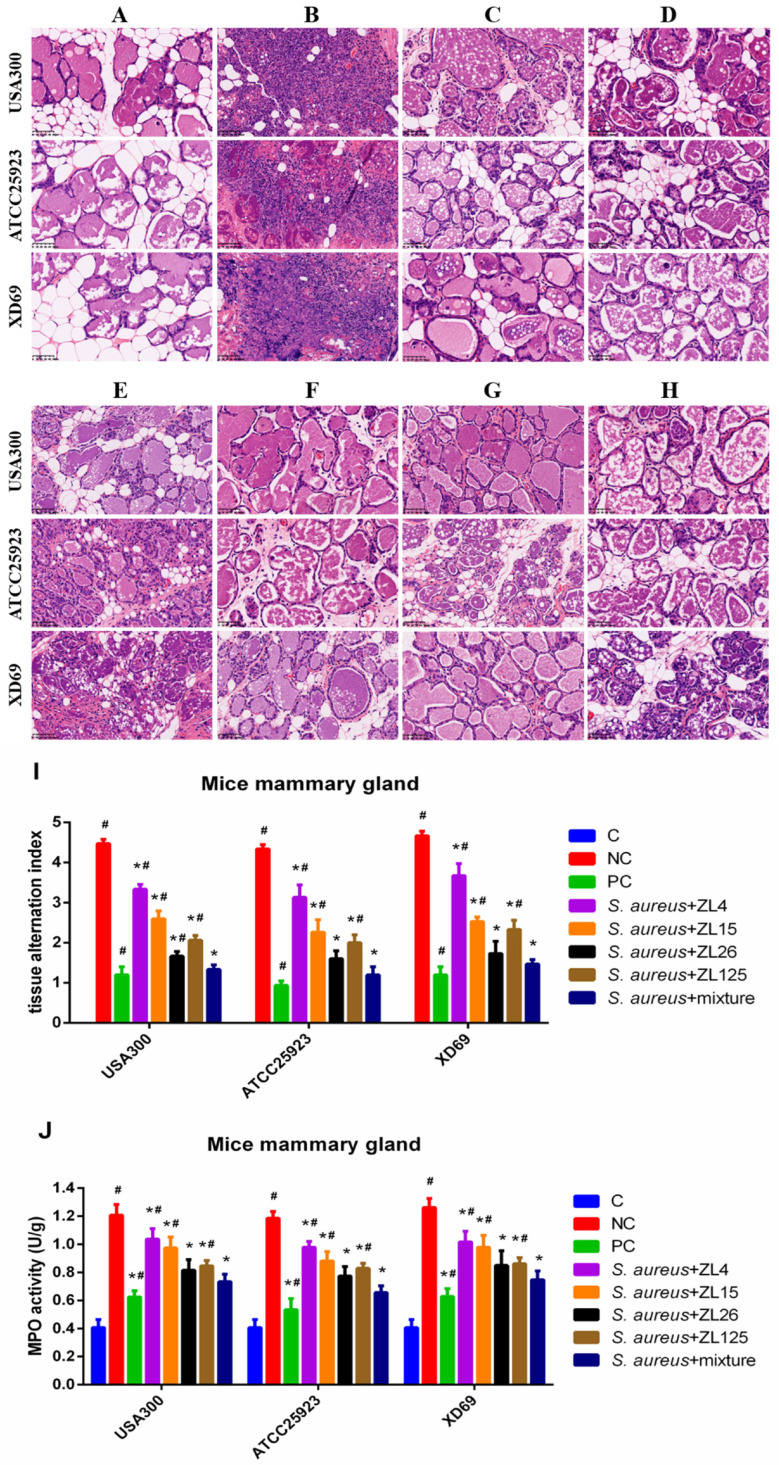
Functions of scFvs on histopathological changes in *S. aureus*-induced mammary gland tissues by H & E staining. (**A**) Control group; (**B**) negative control group; (**C**) positive control group; (**D**) scFvs mixture group; (**E**–**H**) single scFv group (ZL4, ZL15, ZL26 and ZL125, respectively); (**I**) Tissue alteration index in mammary gland tissues; (**J**) MPO activity assay. Red arrow was tissue lesion area (red arrow indicates inflammatory cells infiltration in mammary gland tissues). Data represent mean results ± SD (*n* = 6). # *p* < 0.05 vs. scFvs mixture group. * *p* < 0.05 vs. negative control group.
